# Ultrasonically sculpted virtual relay lens for in situ microimaging

**DOI:** 10.1038/s41377-019-0173-7

**Published:** 2019-07-17

**Authors:** Matteo Giuseppe Scopelliti, Maysamreza Chamanzar

**Affiliations:** 1 Department of Electrical and Computer Engineering, Carnegie Mellon University, 5000 Forbes Avenue, Pittsburgh, PA 15213 USA; 2 Carnegie Mellon Neuroscience Institute, Carnegie Mellon University, 5000 Forbes Avenue, Pittsburgh, PA 15213 USA; 3 Center for the Neural Basis of Cognition, Carnegie Mellon University, 4400 Forbes Avenue, Pittsburgh, PA 15213 USA

**Keywords:** Imaging and sensing, Microscopy

## Abstract

We demonstrate in situ non-invasive relay imaging through a medium without inserting physical optical components. We show that a virtual optical graded-index (GRIN) lens can be sculpted in the medium using in situ reconfigurable ultrasonic interference patterns to relay images through the medium. Ultrasonic wave patterns change the local density of the medium to sculpt a graded refractive index pattern normal to the direction of light propagation, which modulates the phase front of light, causing it to focus within the medium and effectively creating a virtual relay lens. We demonstrate the in situ relay imaging and resolving of small features (22 µm) through a turbid medium (optical thickness = 5.7 times the scattering mean free path), which is normally opaque. The focal distance and the numerical aperture of the sculpted optical GRIN lens can be tuned by changing the ultrasonic wave parameters. As an example, we experimentally demonstrate that the axial focal distance can be continuously scanned over a depth of 5.4 mm in the modulated medium and that the numerical aperture can be tuned up to 21.5%. The interaction of ultrasonic waves and light can be mediated through different physical media, including turbid media, such as biological tissue, in which the ultrasonically sculpted GRIN lens can be used for relaying images of the underlying structures through the turbid medium, thus providing a potential alternative to implanting invasive endoscopes.

## Using sound to sculpt non-invasive imaging lenses

Ultrasound waves can be used to shape a tunable pressure column within a medium that guides light like a lens, providing an alternative to invasive, implanted imaging techniques. Matteo Giuseppe Scopelliti and Maysamreza Chamanzar of Carnegie Mellon University, USA, demonstrated that details of a micro-sized object can be clearly focused through a turbid medium using an ultrasonic transducer. The object could not be seen with an external microscope when ultrasound was turned off. It could be clearly imaged, however, when an ultrasound transducer was used to form a tunable pressure column within the turbid medium. The sound waves propagating through the medium change its density and refractive index and create a column that focuses the light. The technique shows potential for non-invasive imaging and therapeutic light delivery in biological tissues.

## Introduction

Non-invasive propagation of electromagnetic waves in different frequency bands (from X-rays to radio frequency waves and light waves in the visible and near-infrared spectrum) through biological tissue has been widely utilized to access different parts of the body. In particular, light-based methods are now widely used for functional and structural medical imaging as well as therapeutic interventions, such as photodynamic therapy (PDT) of malignant tumors and optogenetic stimulation of neurons^1–3^.

However, scattering of light within biological tissue limits the depth and the resolution of optical methods, especially in the visible and near-infrared range of the spectrum^4–6^. Biological tissue is composed of a heterogeneous mix of large particles, such as nuclei and organelles, or small structures, such as macromolecular aggregates and membranes^7^. When the size of the scattering particles is comparable to the wavelength of light, Mie scattering dominates, whereas when the size is smaller than the wavelength of light, Rayleigh scattering is predominant^8^. As a result, when a collimated or a focused beam of light enters a turbid medium, such as biological tissue, it will be dispersed due to scattering. Therefore, the intensity of light, defined as the power per unit area (mW mm^−2^), rapidly drops, and the spatial resolution degrades with increasing number of scattering events along the beam path. Increasing the input power to compensate for the loss of intensity at the target location will risk photothermal damaging of the tissue at the surface and results in a strong background noise in imaging applications, such as fluorescent microscopy, as well as stimulation of unwanted targets in optogenetic and therapeutic interventions, further deteriorating the spatial resolution.

To address these issues, implantable optical microendoscopes and miniscopes have been devised to guide and collect light through tissue at depth and reach target regions of interest, while maintaining the desired level of intensity and resolution^9–11^. A key component of these miniaturized microscopes is a graded-index (GRIN) lens that relays the optical image. For example, small microendoscopes, equipped with a 0.5–1 mm diameter implantable GRIN lens, have been recently used for one-photon (1 P) calcium imaging in awake free-roaming rodents^12,13^.

These optical implants enable access to deep tissue, but their invasive nature belies the non-invasive promise of optical techniques. In this paper, we discuss a radical approach to use the target medium itself to sculpt and shape a virtual GRIN lens using ultrasonic waves that can propagate through the medium non-invasively, thus providing an alternative to implanting an invasive, bulky physical GRIN lens. In contrast to physical GRIN lens implants that are limited to imaging from fixed positions in tissue, the ultrasonically sculpted optical relay lenses can be reconfigured dynamically to scan and image a 3D volume non-invasively, thus preventing an inflammatory tissue response that would happen when repositioning a physical GRIN lens in the tissue.

Acousto-optic modulators, tunable gratings, and tunable acoustic gradient lenses have been reported in literature and widely used in imaging applications^14–17^. In all of these techniques, a specific material such as a crystal or an oil with a high elasto-optic coefficient is used, in which the refractive index of the material is modulated using ultrasonic waves and, as a result, the beam of light is modulated and shaped before it is launched into the target medium. All of these techniques, similar to any other method based on external optics, suffer from the limited penetration depth in a turbid medium^18^. In other words, an externally patterned beam of light will be dispersed while propagating through the turbid medium. Using such external optics techniques, light can be delivered or collected only from shallow depths in turbid media, such as biological tissues. The novelty of the presented approach, which distinguishes it from traditional acousto-optic technologies, is that the target medium itself mediates the interaction of light and ultrasound; therefore, light is modulated in situ and photons are kept confined and relayed from the depth of the target medium. We have recently shown that in situ virtual steerable optical waveguides can be sculpted using non-invasive ultrasonic waves for light delivery to the depth of scattering media such as brain tissue^19,20^. In this paper, we show that in situ virtual relay lenses can be sculpted in the medium using ultrasound.

## Results

### Ultrasonic waves create a graded refractive index profile in the medium

**Fig. 1:**
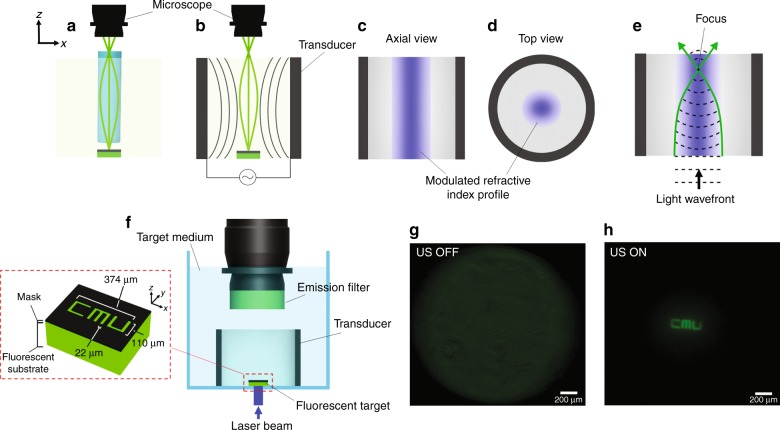
In situ imaging using ultrasonically sculpted virtual relay lens. **a** Schematic of a traditional GRIN lens used as an endoscope inserted in the target medium to relay the image of a target object to an external microscope; **b** ultrasonic waves sculpt a virtual GRIN lens in the medium, effectively creating a relay lens. **c** Axial; and **d** cross-sectional profiles of the ultrasonically modulated refractive index profile in the medium. **e** The input light wave experiences a radially-varying phase delay because of the refractive index profile, which is maximum at the center; as a result, the beam of light will adiabatically converge to a central focal spot. **f** Schematic of the experimental setup used for demonstrating the relay imaging capability of the virtual GRIN lens; inset: illustration of the target object (not to scale), which is placed 29 mm deep into the medium from the focal plane of the external microscope. **g** Out-of-focus image of the target object when ultrasound is off. **h** When ultrasound is turned on, the image of the target object relayed through the medium and resolved by the external microscope. The exposure time used for acquiring Fig. 1g is 5-folds higher than the value used for Fig. 1h

The concept is schematically illustrated in Fig. 1 in comparison with the application of a traditional relay lens. In conventional microendoscopy, a cylindrical GRIN lens is inserted into the medium (e.g., tissue) to relay the image of the target to outside the medium^21–23^ (Fig. 1a). In the presented method, ultrasonic waves sculpt a virtual GRIN lens directly into the medium (Fig. 1b).

To understand the principle of operation, we note that ultrasonic waves launched by a piezoelectric transducer generate pressure waves into the medium. As these waves propagate through the medium, they modulate its density and hence its local refractive index. In a cylindrical geometry, the high-pressure column at the center generates a refractive index modulation profile that has a peak at the same center and then decays radially (Fig. 1c). This in situ sculpted refractive index profile is almost uniform along the axial direction and mainly varies along the radial direction. This radially-changing refractive index profile forms an in situ virtual graded-index (GRIN) waveguide with a circular cross-section (Fig. 1d), similar to a GRIN fiber but with a slightly different refractive index profile. Such a GRIN waveguide confines and guides light and at the same time adiabatically focuses light because the phase front of a uniform plane wave passing through such a refractive index profile is radially modulated. At the center, with the highest refractive index, the phase velocity *v* (i.e., *v* *=* *c/n(r)*, where *c* is the speed of light and *n(r)* is the refractive index of the medium at each radial location, *r*) is the lowest, and it gradually increases in the radial direction. Therefore, the central part of the optical beam experiences a larger phase delay compared to the rest as it propagates through the medium. This modulated phase front will cause the beam of light to adiabatically converge to a central focal spot (Fig. 1e). If the axial length of the GRIN waveguide is chosen properly, it will act as a GRIN lens.

This is similar to what happens in a physical GRIN lens such as an external tunable acoustic gradient-index lens^9,17^. However, here we form and sculpt a virtual GRIN lens in situ, i.e., inside the target medium.

To demonstrate the feasibility of this method, we imaged a fluorescent object as shown in Fig. 1f (see Materials and Methods). Figure 1g shows the completely unresolved image of the target when the ultrasonic relay lens is turned off (i.e., US OFF). The top microscope assembly images the plane on top of the transducer (29 mm away from the target object). When ultrasound is off, the image of the target is not relayed, and as a result, the top microscope cannot resolve the image of the target and captures a completely blurred out-of-focus image (Fig. 1g). When the virtual GRIN lens is formed by using ultrasound at *f*_res_ = 832 kHz, the image can be completely resolved (Fig. 1h), demonstrating that the in situ ultrasonically formed GRIN lens can relay the image through the medium. This technique works in any compressible medium that shows low acoustic propagation loss. It is worth noting that the transducer-medium system has multiple resonance modes, each occurring at a different frequency. These different resonance modes possess different pressure patterns (different amplitudes and spatial configurations).

To describe the modulating effect of the ultrasonic wave generated in the transducer cavity on an optical plane wave, we take a combined approach of simulation and experiment.

**Fig. 2:**
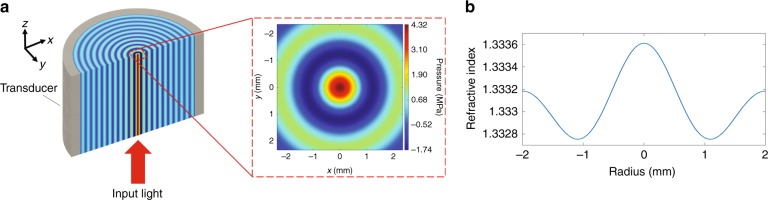
Local pressure modulates the refractive index. **a** Example of a standing pressure wave profile of a resonance mode of an ultrasonic cavity with a diameter of 38 mm formed by a piezoelectric cylinder with a wall thickness of 3 mm, filled with water. The inset shows a 2D cross-section of the pressure. **b** Cross-section of the induced refractive index profile in the medium as a result of the ultrasonic standing wave

In practice, an optical plane wave can be realized by collimating a laser beam, which usually has a cylindrically-symmetric cross-section. To modulate the phase front of such a symmetric beam, we need to form cylindrical interference patterns of ultrasonic waves, so that the phase front is modulated along the radial direction with no azimuthal variation, ultimately resulting in a virtual GRIN lens. Therefore, we used a cylindrical ultrasonic cavity parallel to the direction of light propagation (Fig. 2a). This cavity supports discrete well-defined resonance acoustic modes in the form of standing waves. While the locations of nodes and antinodes are fixed along the radial direction, the antinode amplitude oscillates in time. The virtual GRIN lens is formed only during the positive half cycles. Figure 2a shows the ultrasonic pressure field inside a cylindrical cavity with a diameter of 38 mm at the resonant frequency of *f*_res_ = 832 kHz when the medium is water, at the peak of the positive half period. Ultrasonic waves need to be injected into this cavity using an external source of pressure waves. In practice, we can use a piezoelectric cylinder as the outer wall of the ultrasonic cavity to generate the ultrasonic pressure waves inside the medium. When an electric potential difference is applied to the inner and outer walls of the piezoelectric transducer, it vibrates at the thickness mode. In particular, the input alternating electric field generated by the electric potential applied across the transducer causes its structure to vibrate due to its piezoelectric material property. The periodic vibrational displacements of the transducer walls result in the vibration of the medium surrounded by the piezoelectric transducer. As a result, according to the Helmholtz equation (see Materials and Methods), an ultrasonic pressure wave is launched into the medium that propagates radially. In steady state, this ultrasonic pressure wave forms a standing wave that changes radially.

For example, if we consider a piezoelectric cylinder with a thickness of 3 mm immersed in water, we can induce a maximum ultrasonic pressure of *P*_*max*_ = 4.32 MPa in the center of the cavity, when driven by a sinusoidal electrical signal with an amplitude of *V* = 34 V and a frequency of *f*_*res*_ = 832 kHz. The central positive pressure peak is surrounded by a negative pressure ring (Fig. 2a).

The presented method is general and can be extended to geometries other than cylindrical. For example, multiple ultrasonic transducers can be used in the form of an array to generate complex pressure patterns to modulate the refractive index of the medium and form complex relay lenses^20^.

The refractive index *(n)* of any medium is a function of its density (*⍴*)^24^. In this work, we assume a linear relation between *n* and *ρ* since the change of the medium density is small compared to its static background density, and therefore, we can use a linearized version of the Lorentz–Lorenz equation^25^.

As the ultrasonic waves propagate through the medium, they modulate its density and hence its local refractive index; the medium is compressed in the high-pressure regions, resulting in a higher density, while it is rarefied in the negative pressure areas, where the local density is reduced. As a result, the pressure standing wave creates a local refractive index contrast^26,27^ (defined as the difference in the index of refraction values between the locations of positive pressure peaks and negative pressure troughs) in the medium within the cylindrical cavity of the transducer, whose cross-sectional profile is shown in Fig. 2b. This refractive index profile corresponds to the zeroth order Bessel mode of the ultrasonic cavity. The refractive index profile can be calculated from the simulated pressure profile using the following linear relation:

1 $$n(r) = 1.333 + kP(r)$$

where *r* is the radial distance, *n(r)* is the radial refractive index, *r* is the radial distance, 1.333 is the refractive index of water at room temperature (25 °C) in the visible optical wavelength range, *P(r)* is the pressure (in bar unit) inside the transducer, and *k* = 1.402 × 10^−5^ bar^−1^ is an empirical coefficient. This empirical relationship is extracted from the experimental results reported in literature^28^, where the refractive index of the background medium is measured under different pressure levels using a precision interferometer with a sensitivity of 0.00001 refractive index unit (RIU).

Equation 1 shows that the refractive index profile follows the pressure profile of the standing wave inside the cavity; thus, the maximum refractive index contrast is achieved between the central region and the first negative pressure ring of the Bessel function. For the specific case discussed earlier, the maximum refractive index contrast is *Δn* = 8.5 × 10^−4^. The refractive index profile gradually rolls off along the radial direction when moving away from the center of the ultrasonic cavity, similar to the parabolic decaying profile of the refractive index in a GRIN lens. This in situ sculpted refractive index profile is assumed to be uniform along the axial direction and mainly varies along the radial direction.

However, in contrast to a traditional GRIN lens, the induced refractive index profile for the central lobe is not perfectly parabolic, and therefore, not all the light rays will be directed exactly at the same axial focal position, resulting in spherical aberration. Consequently, photons from different depths within the sample will be captured at the image plane simultaneously. Therefore, the captured images will be blurred; this results in lower contrast and sharpness. It should be noted that the spherical aberration is not an inherent problem of the presented technique since it can be avoided by designing the ultrasonic pattern to produce a parabolic refractive index profile using a phased array or, alternatively, the aberration can be corrected using computational deblurring and deconvolution techniques^29^.

To represent the light path when it travels through such an ultrasonically modulated medium, we need to solve Maxwell’s equations^30^ in a medium where the refractive index changes only along the radial direction as *n*(*r*), because the ultrasonic cavity is axisymmetric and we can also assume that the axial variation of refractive index is negligible, i.e., dn(z)/dz = 0 (see Materials and Methods). Since the wavelength of ultrasound (i.e., λ_s_ = c_s_/f_res_ ≈ 1.78 mm, where the speed of sound in water c_s_ = 1487 m s^−1^ and *f*_*res*_ *=* 832 kHz) is much larger than the wavelength of light (i.e., *λ*_*light*_ = 450–700 nm), and since light propagates over a long distance in the modulated medium (30 mm), solving Maxwell’s equations becomes a numerically intractable problem in such a large simulation domain. Therefore, we employ a numerical method based on solving the Eikonal equation under paraxial approximation (see Materials and Methods).

**Fig. 3:**
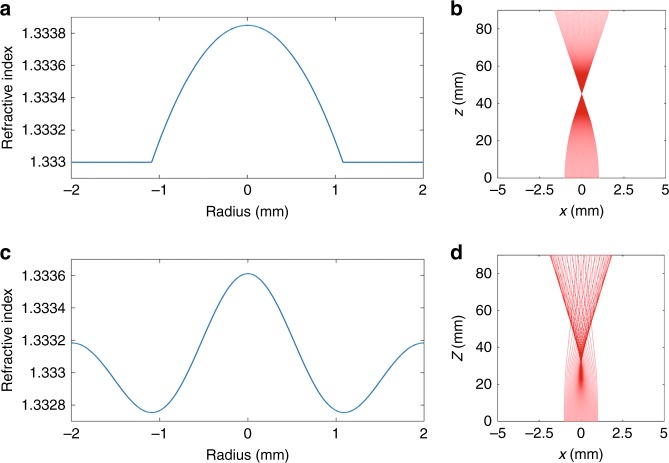
Optical simulations. **a** Parabolic refractive index profile of a conventional GRIN lens with a maximum contrast of Δ*n* = 8.5 × 10^−4^ and **b** the simulated optical beam profile along the 30 mm length of the GRIN lens. **c** Ultrasonically sculpted refractive index profile (zeroth-order Bessel function) with the maximum contrast of Δ*n* = 8.5 × 10^−4^ and **d** the simulated optical beam profile formed inside a 30 mm long piezoelectric transducer

As an example, we have compared the trajectory of light in a conventional GRIN lens to that of our ultrasonically sculpted GRIN lens (Fig. 3). Figure 3a shows the typical parabolic refractive index profile of a conventional GRIN lens, and Fig. 3b shows the focused beam of light passing through it. Figure 3c, d shows the refractive index profile generated by the standing ultrasonic wave and the corresponding trajectory of light, respectively. In both cases, we assumed a maximum refractive index contrast of *Δn* = 8.5 × 10^−4^. The interaction length is 30 mm both for the physical GRIN lens and the ultrasonically sculpted one.

Although the refractive index contrast is the same for both cases, the slightly different profile shapes are responsible for the different focal distances and depth of focuses (DOFs). In particular, the focal point is located at *z* = 45.1 mm for the parabolic profile (Fig. 3b) and at *z* = 29.67 mm for the ultrasonically formed GRIN lens (Fig. 3d). The accuracy of these results is substantiated by comparison with the experimental results in the rest of the paper.

**Fig. 4:**
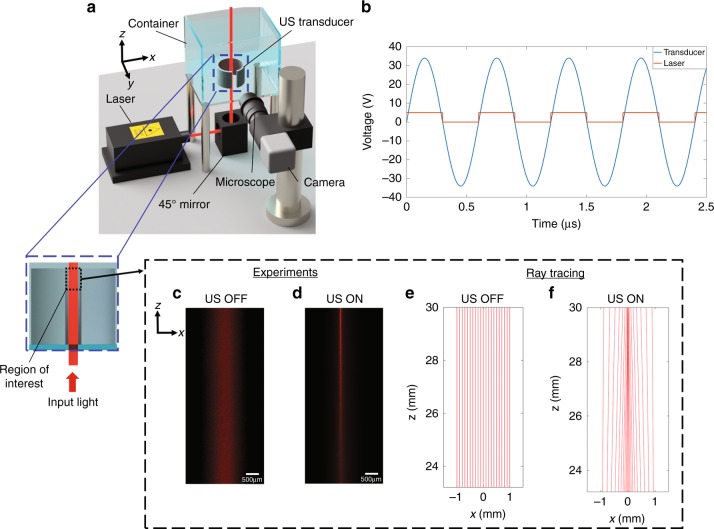
Experimental demonstration of focusing. **a** Schematic of the optical characterization setup; inset: region of interest for experiments. **b** The pulsed laser is modulated in time to match the positive semi-period of the sinusoidal signal feeding the transducer. **c** Experimental images of the laser beam when the ultrasonic transducer is off and (**d**) when it is turned on (f_res_ = 832 kHz, V = 34 V). **e, f** Optical ray tracing simulations of the laser beam for the same frequency and voltages used for the experiments in **c** and **d**

To experimentally demonstrate the in situ ultrasonically sculpted tunable GRIN lenses inside the medium, we built a custom-designed optical characterization setup, shown in Fig. 4a, to laterally image the trajectory of light passing through a medium modulated by ultrasound. In this setup, a collimated laser beam at *λ*_*light*_ *=* 650 nm is vertically directed to a water tank to pass through a cylindrical piezoelectric transducer along the axial direction. We opened a small lateral window in the cylindrical transducer by cutting out a small region of the wall (4 mm arc length), specifically to image the axial trajectory of the optical beam inside the ultrasonic cavity. We verified that this modification does not significantly affect the generation of pressure patterns and, consequently, the beam shaping capability of the transducer. For this experiment, we used a homogeneous scattering solution made of water mixed with a 2 × 10^−4^% concentration of Intralipid emulsion (*I141*, Sigma–Aldrich, Inc., USA). Such a low-scattering medium enables imaging of the axial trajectory of the light beam from the side without causing significant perturbation to the beam profile^31^. The field-of-view (FOV) of our imaging system is 6.8 × 8.3 mm, large enough to image the region of interest for our experiments (inset of Fig. 4a), extending from *z* *=* 23.2 mm to *z* = 30 mm.

In theory, ultrasonic pressure waves can potentially affect the Intralipid droplets in the emulsion and thus change the local scattering properties. The average size of Intralipid droplets is less than 0.5 µm^32,33^; therefore, we expect the radiation force exerted by ultrasound on these droplets be negligible^34^.

We pulsed the laser at the ultrasound frequency and synchronized it with the transducer driving signal, setting the duty cycle at 50%, so that light interacts with the pressure pattern only when the central pressure peak is positive and forms a focusing GRIN lens (Fig. 4b). This process ensures enhanced imaging of the beam by reducing the undesired background that forms by the out-of-focus light during off-cycles and thus increases the contrast.

When ultrasound is off, the laser beam remains collimated along its propagation path (Fig. 4c). When ultrasound is activated (*f*_*res*_ = 832 kHz, *V* = 34 V), pressure waves create the desired refractive index profile inside the ultrasonic cavity, thus focusing the beam of light in the medium (Fig. 4d). Our numerical simulations (Fig. 4e, f) match the experimental results (Fig. 4c, d). The simulation results predict a focal distance of 29.67 mm, which agree very well with the experimental results, i.e., a focal distance of 29.68 mm.

### Reconfigurable ultrasonic wave pattern enables in situ axial scanning of the focal point inside the medium

So far, we have shown that ultrasonic waves can sculpt a GRIN lens in a medium by spatially modulating the local refractive index to define the trajectory of the optical beam path in situ as it propagates through the medium. The in situ virtual lens can be temporally reconfigured by changing the ultrasonic pressure waves inside the medium, which is similar, in concept, to externally tunable ultrasonic lenses, where the axial location of the focal point can be changed dynamically^17^. However, the fundamental difference in this work is the in situ scanning of the axial focus in the target medium. We will show that the numerical aperture (NA), and therefore, the focal distance, which is inversely proportional to NA can be tuned by changing the ultrasonic wave amplitude inside the cavity.

As discussed earlier, the refractive index of the medium is a function of ultrasound pressure amplitude. The maximum refractive index contrast is proportional to the ultrasound peak intensity, which in turn is a function of the input electric potential amplitude applied to the ultrasonic transducer. Therefore, by changing the input electric potential, the maximum refractive index contrast is changed, and the focal depth can be adjusted.

**Fig. 5:**
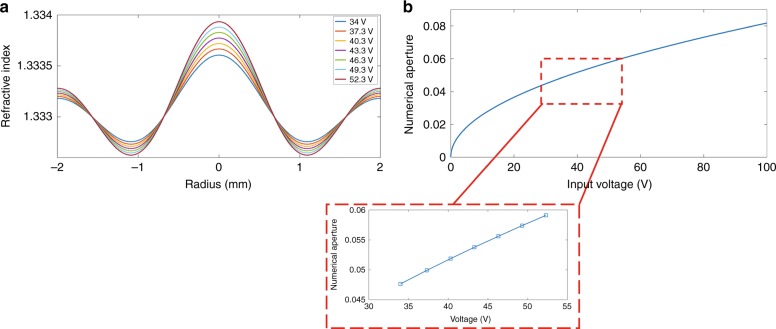
Reconfiguring the refractive index profile. **a** Peaks and troughs of the ultrasonically sculpted refractive index profile change with the applied input electric potential at f_res_ = 832 kHz. **b** Increasing the intensity of the ultrasonic waves results in an increase of the NA of the virtual ultrasonically sculpted GRIN lens. Inset: variation of the NA for the input electric voltages used in our experiments

When the input electric potential is increased, the magnitude of pressure at the antinodes of the stationary pressure wave increases, thus increasing the maximum refractive index contrast (Fig. 5a). Therefore, the optical phase front is modulated more strongly, and a stronger GRIN lens is formed since increasing the refractive index contrast results in an increase of the numerical aperture. In a GRIN lens, the numerical aperture is defined as:

2 $${\mathbf{NA}} = \sqrt {{\mathbf{n}}_{{{{\mathrm{max}}}}}^2 - {\mathbf{n}}_{{{{\mathrm{min}}}}}^2}$$

Figure 5b shows the NA as a function of the input electric potential. For the voltages employed in our experiments, ranging from 34 V to 52.3 V (inset of Fig. 5b), the NA varies from 0.04765 to 0.0591. At *f*_*res*_ = 832 kHz, a 21.3 V increase in the electrical potential results in an estimated 21.5% increase of the NA.

**Fig. 6:**
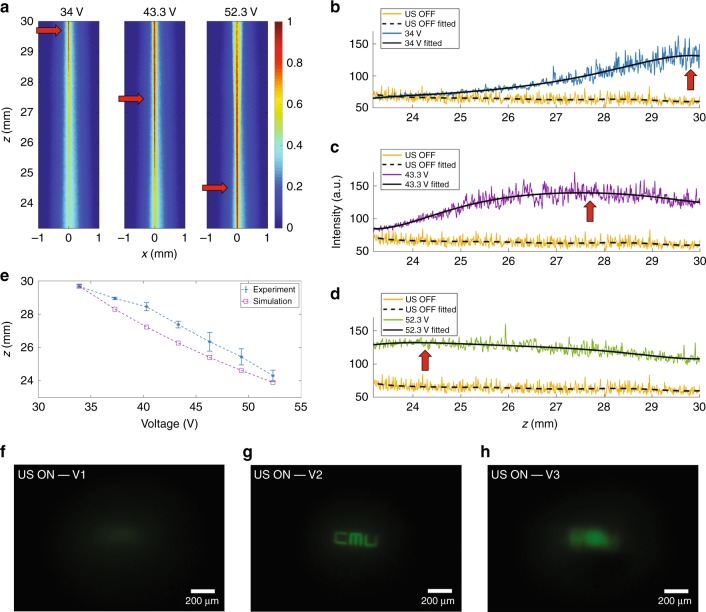
Scanning through the depth of the medium. **a** Experimental images of the axial profiles of the optical beam inside the ultrasonic cavity for three different input voltages at the resonant frequency f_res_ = 832 kHz, showing the axial foci at the top, in the middle, and at the bottom (indicated by red arrows). Raw data and fitted curves showing the intensity profiles of light for the three images in Fig. 5a when the transducer is off and when it is on, driven at **b** 34 V, **c** 43.3 V, and **d** 52.3 V. The maximum intensities of the fitted profiles are indicated by red arrows. **e** Comparison between the simulated axial locations of the foci and the experimental results. Changing the input drive voltage enables axial scanning: **f** at V1 = 40.3 V, the target is out of focus, **g** at V2 = 46.3 V, the image is clearly resolved, and **h** a further increase in voltage to V3 = 52.3 V results in a newly out-of-focus image

Therefore, by continuously increasing the electric potential applied to the transducer, we can scan the focal point along the *z*-axis. We performed experiments using the setup shown in Fig. 4a to demonstrate scanning of the focal distance. Figure 6a shows the axial beam profiles imaged laterally through the window opening on the side of the transducer when it is driven at 34, 43.3, and 52.3 V. From these images, we can clearly see that the highest intensity point is moved downwards along the axial direction as the input electric potential and consequently the input intensity of the ultrasonic waves is increased. The axial intensity profile for each case is plotted by obtaining the maximum intensity value for each radial cross-section of the captured images at each location along the *z*-axis. Figure 6b–d shows the optical beam intensity profiles when ultrasound is off (baseline) compared with the case when ultrasound is on for the three different input electric potentials.

The baseline intensity slightly decreases along the axial direction due to the low scattering in the medium. A sixth order polynomial fit was used to extract the envelopes (black solid lines), using which we can obtain the location of maximum intensity that represents the focal point. The formation of a focal point for *V* = 34 V is shown in Fig. 6b. The intensity of the beam at 23.2 mm is comparable to the baseline, i.e., to the US OFF case, but gradually increases until reaching a maximum at *z* = 29.68 ± 0.1 mm, after which the intensity decreases again.

The intensity profiles for other voltages of 43.3 V and 52.3 V are plotted in Fig. 6c, d, respectively. The axial focus is at *z* = 27.38 ± 0.2 mm for the 43.3 V input electric potential and at *z* = 24.3 ± 0.33 mm for the 52.3 V input electric potential. A total voltage increment of 18.3 V is enough to change the focal length of the virtual sculpted lens by 5.4 mm, equivalent to 18% of the initial focal length. To establish a quantitative correlation between the input electric potential and the axial position of the focus, we performed numerical simulations to calculate the focal distances for seven different input electrical potentials starting from 34 V with step sizes of 3 V. The frequency was fixed at *f*_*res*_ = 832 kHz. For each ray tracing simulation, we measured the distance between the input plane at the bottom of the transducer (i.e., the reference point at *z* = 0 mm) and the location of each focal point. We did not increase the input electric potential beyond 52.3 V, because the prolonged exposure to high-intensity ultrasound can elevate the temperature of the medium and thus change the resonance of the ultrasonic waves, which results in an unstable virtual GRIN lens. To mitigate this issue, ultrasound can be modulated so that it does not stay on continuously for a long period of time. Moreover, for the particular type of transducer that we have used in our experiments, the threshold of damage for the piezoelectric ceramic is ~75 V, which serves as the absolute maximum possible input electric potential in this case. We found that a voltage range of 34–52.3 V is appropriate for a rather large axial scanning range (i.e., 5.4 mm) without any severe heating or instability issues.

Figure 6e shows a comparison between the experimentally measured focal distances (solid line) and simulation results (dashed line). The discrepancy between the experimental and the simulation results (denoted by *Δz*) is small. This discrepancy is mainly due to the axial variation of refractive index, temperature drifts and other non-idealities in the experiments. For the three cases, where the electric potentials applied to the transducer are 34, 43.3, and 52.3 V, the *Δz* values are 0.08, 1.11 and 0.4 mm, respectively.

The axial scanning capability of the presented method can be used to image objects at different depths within the medium. We imaged the same fluorescent target object that was used to obtain the results in Fig. 1 under different input electric potentials. For a 40.3 V input signal, the focal plane of the virtual GRIN lens was formed below the surface of the target, and the image was, therefore, out of focus (Fig. 6f). For a 46.3 V input electrical signal, the image was in focus (Fig. 6g), and at a higher input electric potential of 52.3 V, the relay lens imaged a surface above the target object, and therefore, the image was out of focus again (Fig. 6h).

### Ultrasound enables optical relay imaging through scattering media

In the previous sections, we presented results obtained in an optically transparent medium, i.e., water with only a negligible concentration of scattering agents (Intralipid), to enable lateral imaging of the formed optical beam. One of the potential applications of the proposed technique is imaging through a biological medium by sculpting an in situ virtual GRIN lens in the tissue, which can potentially replace the bulky and invasive GRIN relay lenses commonly used in microendoscopes. In this way, our in situ GRIN lens can extend the reach and flexibility of external optics for accessing otherwise inaccessible regions within the biological media.

**Fig. 7:**
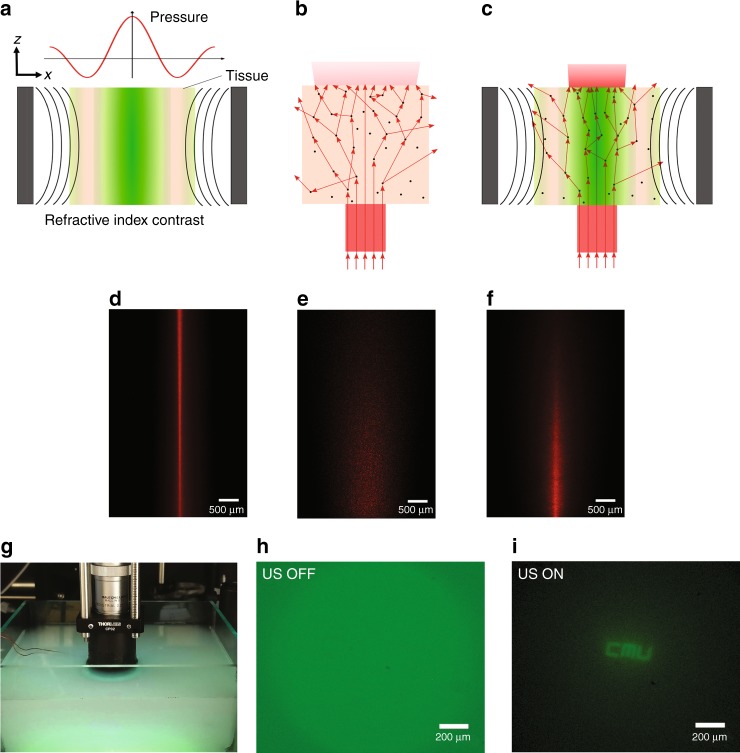
Virtual relay imaging through scattering media. **a** A local refractive index contrast is generated in the tissue by US pressure waves. **b** A collimated beam of light is expanded as a result of scattering in the tissue. **c** Light is confined in the high refractive index region, counterbalancing the effect of scattering in the tissue. Experimental results showing: **d** laser beam focused by ultrasound in a minimally scattering tissue phantom; **e** dispersion and attenuation of the laser beam passing through a scattering tissue phantom without ultrasound; **f** laser beam focused by ultrasound in a scattering tissue. **g** The measurement setup to demonstrate the relaying effect of the virtual ultrasonically sculpted GRIN lens through a deep scattering medium, which is normally opaque in the visible range. **h** When the transducer is OFF, the image is out of focus, and the strong background caused by scattering is evident. **i** When the transducer is driven at 46.3 V, the image of the target object is relayed through the 29 mm depth of the turbid medium and is clearly resolved. The depth of this turbid medium corresponds to an optical thickness of OT = 5.7 MFP. The exposure time used for acquiring Fig. 7h was three times higher than the exposure time used for Fig. 7i

Ultrasonic waves undergo minimal attenuation (~0.3–0.6 dB cm^−1^ MHz^−1^ (ref. ^35^)) when propagating through biological tissue; therefore, ultrasound can be used to sculpt the presented in situ GRIN lenses inside biological tissue. The standing pressure wave will change the local density of the biological tissue medium, and it can induce a refractive index profile in the biological tissue to sculpt an in situ GRIN lens (Fig. 7a).

When a collimated beam of light propagates through biological tissue, it is dispersed due to scattering of photons (Fig. 7b); as a result, the output beam will be wider, and its intensity will be lower. However, when the refractive index profile of a GRIN lens is ultrasonically sculpted into the tissue, the focusing effect of the virtual relay lens will compete against the dispersive effect of scattering, resulting in an effectively confined and narrower beam of light (Fig. 7c).

Figure 7d shows the lateral image of the light beam confined and focused by ultrasound in a homogeneous transparent medium with negligible scattering (i.e., tissue phantom made of agar 2%).

To experimentally observe the competition effect, we prepared a thick (6 mm) scattering tissue phantom made of 2% agar + 0.1% Intralipid. The reduced scattering coefficient was measured to be *μ*_*s*_*’* = 1.35 cm^−1^ using the Oblique Incidence Reflectometry method^36^. The optical thickness (OT) of this phantom can be estimated to be 8.1 MFP. When the laser beam passes through the scattering tissue phantom without the ultrasonic modulation (Fig. 7e), it is gradually scattered as it propagates; consequently, the beam widens, and the intensity drops along the axial direction. When the same scattering medium is modulated by ultrasound and a GRIN lens is formed inside the medium, light is effectively confined and focused (Fig. 7f).

The sculpted GRIN lens in a scattering turbid medium is not as perfect as a virtual lens in a transparent medium, but it can still function as an effective relay lens. As shown in Fig. 7g, we immersed the fluorescent target object (that we used in our previous experiments in a non-scattering medium) 29 mm deep into a scattering medium made of water + 0.025% Intralipid (*μ*_*s*_*’* *=* 0.196 cm^−1^ ^37,38^, *g* ≈ 0.9 (ref. ^39^)). While the reduced scattering coefficient might seem small in this case, the rather long imaging depth (i.e., 29 mm) corresponds to an optical thickness of *OT* = 5.7 MFP, meaning that light will undergo multiple scattering events (i.e., an average of about 6 scattering events) as it travels from the target object to the surface of the tissue phantom. Similar to the characterization setup used for the previous relay imaging experiments, the top microscope assembly images the plane at the top edge of the ultrasonic transducer. When ultrasound is off (Fig. 7h), the captured image shows a strong background caused by light scattering overlaid by a higher intensity circular region in the center, corresponding to the out-of-focus image of the fluorescent target passing through the scattering medium. Driving the ultrasound at *f*_*res*_ = 832 kHz with an amplitude of 46.3 V, we can clearly relay the image of the target object through the scattering tissue phantom from a depth of 29 mm to the surface and capture it using the external optical microscope (Fig. 7i).

## Discussion

Ultrasonic waves can be used to sculpt in situ virtual, reconfigurable GRIN lenses capable of non-invasively modulating and shaping the wavefront of light within a medium, enabling non-invasive relay imaging. The virtual GRIN lens sculpted in the medium can be reconfigured by changing the parameters of ultrasonic waves. As an example, we demonstrated that the focal distance can be continuously scanned along the axial direction in a medium with negligible optical scattering (water + 4 × 10^−4^% Intralipid) over a range of 5.4 mm. In addition to changing the focal distance, the numerical aperture of the ultrasonically sculpted GRIN lens can also be tuned by varying the ultrasound intensity. We demonstrated that by varying the input electric potential in the range of 34–52.3 V at a fixed resonant frequency of 832 kHz, the numerical aperture can be tuned up to 21.5% of the starting value (NA = 0.04765). Both the axial scanning range and the numerical aperture are functions of the ultrasound intensity, the elasto-optic properties of the medium, and ultrasound resonant frequency, as well as the interaction length of ultrasound and light in the modulated medium within the transducer. It should be noted that in the ultrasonically sculpted GRIN lens, the axial scanning range and the numerical aperture are coupled to each other and cannot be independently changed. However, using the various degrees of freedom mentioned above, one can optimize the parameters to achieve the desired axial scanning range as well as the desired numerical aperture.

The virtual ultrasonically sculpted GRIN lens presented in this work can be used for imaging through a medium without inserting an invasive conventional physical GRIN lens, which is a unique advantage of the presented technique that enables relay imaging through the medium non-invasively. Ultrasonic waves can penetrate into any compressible medium that exhibits low acoustic propagation loss to form a virtual GRIN lens inside the medium. We have demonstrated that the ultrasonic virtual GRIN lens can relay the image of a fluorescent target object (with feature sizes as small as 22 µm) through 29 mm depth of the target medium. We experimentally demonstrated that the non-invasive relay imaging can be performed both in transparent and scattering media. We used a turbid medium consisting of Intralipid as the scattering agent with an optical thickness of *OT* = 5.7 MFP, as a typical example of a medium in which photons undergo an average of about 6 scattering events. The quality of the image and the depth of penetration are functions of the ultrasonic wave parameters such as intensity, frequency, and pattern of ultrasound. Further characterization and benchmarking of this technique can be carried out by optimizing the sculpted refractive index profile in different turbid media. Of course, the feasible range of the parameters for ultrasound would depend on the specific application.

For example, using this technique for imaging in biological tissue requires that ultrasound parameters fall within the safe range for the tissue, since high-intensity ultrasound can cause thermal and mechanical damage to the tissue, including generating localized heat and cavitation^40^.

According to the Food and Drug Administration (FDA) regulations for the biosafety of ultrasound, the spatial-peak temporal-average intensity (*I*_*SPTA*_) and spatial-peak pulse-average intensity (*I*_*SPPA*_) must be less than 720 mW cm^−2^ and 190 W cm^−2^, respectively^41^. Our technology can comply with both of these intensity limits by properly choosing the ultrasound parameters. In our method, we can pulse and synchronize the ultrasonic waves and laser pulses so that their interaction happens only when the pressure is at the positive peak value, thus minimizing the average acoustic power transferred to the tissue^19,20^.

As an example, if the virtual in situ lens is to capture 10 images per second, we can set the pulse repetition frequency (*PRF*) of the electrical signal driving the ultrasonic transducer to be 10 Hz with 75 cycles per pulse at a frequency of 832 kHz. In this way, considering an estimated peak pressure (derated by 0.3 cm^−1^ MHz^−1^) of *P*_*.3*_ = 5.29 MPa at the center of the transducer (for imaging experiments at 46.3 V input drive signal) and a typical soft tissue propagation medium, with an acoustic impedance of *z* = 1.63 × 10^6^ kg m^−2^ s (ref. ^42^), the I_SPTA_ is 690 mW cm^−2^, less than the safety threshold of 720 mW cm^−2^ imposed by the FDA.

It is also worth noting that the pressure values reported in this work are specific to the geometry of the transducer and the signal frequency. For example, if the same transducer were 41 mm long (the one employed for this work was 30 mm), we would only need 2.23 MPa, which is 48% less than the pressure we used in Fig. 2a to focus light in situ at a focal distance of 40.75 mm. In this case, the *I*_*SPPA*_ is 113.1 W cm^−2^, which is within the safe range as defined by the FDA, i.e., *I*_*SPPA*_ < 190 W cm^−2^.

**Fig. 8:**
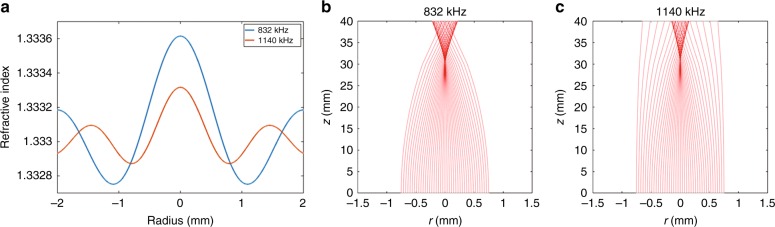
Effect of frequency and ultrasound pressure. **a** Comparison between the ultrasonically sculpted refractive index profiles at f_1_ = 832 kHz, for a peak pressure of 4.32 MPa (blue line) and at f_2_ = 1140 kHz, for a peak pressure of 2.25 MPa (orange line). **b, c** Optical ray tracing simulations of light propagating through the two media modulated by the refractive index profiles shown in **a**. In both cases, the focal distance inside the medium is ~30 mm. For the higher frequency ultrasonic waves (at f_2_ = 1140 kHz), the width of the central high refractive index region is smaller compared to the lower frequency ultrasonic waves (at f_2_ = 832 kHz) and therefore, with a lower peak pressure, light can be focused at the same plane

Another option to reduce the maximum amount of needed pressure is to operate at higher frequencies, where the wavelength of ultrasound is shorter and, as a result, the central peak is narrower, which will cause light to focus faster. Figure 8a shows the refractive index profiles sculpted at two frequencies, *f*_*1*_ = 832 kHz and *f*_*2*_ = 1140 kHz. For the higher frequency case (i.e., 1140 kHz), the maximum pressure generated in the transducer is 2.25 MPa, which is 48% less than the pressure required for the lower frequency case as reported in Fig. 2a. The ray tracing simulations (Fig. 8b, c) show that light is focused at a depth of 30 mm for both frequencies. Therefore, by simply increasing the frequency to 1140 kHz, we can focus light in situ at the same location, and the *I*_*SPPA*_ is 115.13 W cm^−2^, which is again within the safe range.

In future, we can optimize and refine this technique depending on the specific application and properties of the tissue. In particular, we can design more sophisticated ultrasonic systems (number of ultrasonic transducer elements, geometry, and material) and optimize the ultrasound parameters (frequency, modulation, and duty cycle) to make sure that our technology meets the safety requirements to prevent damage to the tissue.

We note that the heterogeneity of the biological tissue will affect the sculpted virtual in situ GRIN lens and might hamper its performance. Such adverse effects can potentially be compensated for by using an array of ultrasonic transducers to sculpt the in situ virtual GRIN lens into the tissue, where the parameters of the individual array elements can be adjusted accordingly to counterbalance the effect of heterogeneity in the tissue.

A unique feature of the virtual GRIN lens technique is that light is shaped and focused in situ inside the target medium. Compared to external optics techniques, such as holographic microscopy or imaging using spatial light modulators (SLMs), where light is shaped outside the medium and then is launched to propagate into the target medium, our technique has the important advantage that it can actively tailor the optical beam path inside the medium. This powerful capability can be used to design more sophisticated virtual in situ GRIN lenses with modified refractive index profiles to correct for aberrations and other anomalies to resolve a sharp image of the target.

The presented method can be generalized to an array of ultrasonic transducers to adjust the point spread function of the sculpted virtual GRIN lens in situ, for example, to compensate for the heterogeneity of the medium. Moreover, we have recently demonstrated that using an array of ultrasonic transducers, complex steerable patterns of light can be sculpted in the medium^20^. This technique can be adopted to steer the focal point of the virtual in situ GRIN lens for scanning or to generate multiple parallel focal points for parallel imaging. In addition to imaging, the virtual relay lens can also be used for non-invasive light delivery to the tissue for applications, such as photodynamic therapy and optogenetic stimulation of brain activity. The notion of sculpting and shaping virtual GRIN lenses inside a medium without implanting a physical lens can be combined with existing imaging and light-based techniques and will find intriguing applications in biological imaging and therapeutic intervention, metrology, and related fields.

## Materials and methods

### Modeling and simulation of ultrasonic pressure waves

To model the ultrasonic waves generated by the piezoelectric transducers, we employed a commercial Finite Element Method (FEM) software environment (COMSOL Multiphysics^®^ v. 5.3, COMSOL AB, Stockholm, Sweden). Our model requires solid mechanics analysis, electrostatics, and pressure acoustics to be coupled to properly simulate the pressure distribution generated by the transducer.

In the software, we first solved the Navier’s equation for the piezoelectric structure, and then we used the Gauss law for the electrostatics modeling, and finally solved the Helmholtz equation for the pressure acoustics. The details are summarized in the following equations:

Navier’s equation:

3 $$- \rho \omega ^2{\boldsymbol{u}} - \nabla \cdot {\boldsymbol{\sigma }} = {\boldsymbol{F}}e^{i\varphi }$$

where ω is the temporal frequency, **u** is the displacement field, **σ** is the stress tensor field, **F** is the external volumetric force distributed over the transducer that originates from external loads (such as body loads or gravity), and e^iϕ^ indicates the phase. In our model, we did not assume any initial volumetric force. However, the pressure generated in the medium is physically coupled to the transducer and will act as an external load ***F***, which can be calculated as:

4 $${\boldsymbol{F}} = p_t{\boldsymbol{n}}$$

where p_t_ is the total acoustic pressure in the medium and **n** is the vector that shows the normal to the transducer walls.

Gauss law:

5 $$\nabla \cdot {\boldsymbol{D}} = \rho _f\;{\rm or}\;\nabla \cdot {\boldsymbol{E}} = \frac{\rho }{{{\it{\epsilon }}_0}}$$

where **D** is the electric displacement field, ρ_f_, ρ are the free and total volume charge densities, respectively, **E** is the electric field, and ${\it{\epsilon }}_0$ is the permittivity of vacuum.

Helmholtz equation:

6 $$\nabla \cdot \frac{1}{\rho }\left( {\nabla p_t - q_d} \right) - \frac{{k_{eq}^2p_t}}{\rho } = Q_m$$

where p_t_ is the sum of the perturbation sound pressure p and background pressure p_b_, q_d_ is the dipole source, $k_{eq} = \sqrt {\left( {\frac{\omega }{c}} \right)^2 -\, k_z^2}$ is the equivalent wavenumber, containing both the ordinary wavenumber k as well as the out-of-plane wavenumber k_z_ (set to 0 rad m^−1^ since we are considering the radial propagation of waves), m is the circumferential wavenumber, Q_m_ is the monopole source, ρ is the density, and c is the speed of sound. In the present model, no monopole or dipole sources were included. Therefore, q_d_ = 0 N m^−3^, and Q_m_ = 0 s^−2^.

The simulations were performed in two dimensions (2D); the domain is defined by a cross-section of the cylindrical geometry, assumed to be infinite along the axial direction (*z-*axis). We used a perfectly matched layer (PML) boundary condition for the outer boundaries to prevent any reflections and interference of waves, thus mimicking an infinite medium. We set the freely vibrating boundary condition to the inner and outer walls of the piezoelectric cylinder. The desired electric potential is applied to the outer wall of the transducer, while the inner wall is set as the electric ground. The material and geometrical properties used in the simulations are listed in Table 1.

**Table 1:** Material and geometrical properties used in the FEM simulations

Piezoelectric transducer material	
Material	Piezoelectric PZT—5 A
Cylindrical ultrasonic Transducer	
Inner radius	19 mm
Outer radius	22 mm
Height	30 mm
Simulation domain	
Radius	27 mm
Medium density (water)	1000 kg m^−3^
Speed of sound	1487 m s^−1^
Perfectly matched layer (PML)	
Radius range	27–37 mm

We used a free triangular mesh with different sizes for different domains. We used an average mesh size of 260 μm for the piezoelectric material domain, a 110 μm mesh size for the medium (where the pressure profile is calculated), and an average mesh size of 900 μm in the PML region.

Initially, we solved the set of coupled equations in the eigenfrequency domain to obtain all the eigenmodes of the system. Then, we assumed a time-harmonic input electric potential and solved the same problem in the frequency domain at the sustained cavity resonance modes. This way, we could obtain the pressure amplitudes for the sustained modes of the cavity for any given input voltage.

The pressure profile at any given resonance frequency of interest was then calibrated to match the experiments, given all of the simplifications and assumptions of the simulation model (i.e., 2D approximation, axially infinite structure and no acoustic loss). Finally, the pressure profile is transformed into a refractive index profile (Eq. 1), which is then used as the input to our optical simulation.

### Optical simulations

The refractive index changes smoothly in the ultrasonically modulated medium. Therefore, we can solve the Eikonal equation^43^ to calculate the ray path:

7 $$\frac{{d^2r}}{{dz^2}} = \frac{1}{{n(r)}}\frac{{dn(r)}}{{dr}}$$

The change of refractive index along the radial axis is much smaller than the background refractive index (*Δn* *<* 10^−3^); thus, we can use the paraxial approximation^44^ to simplify the solution to the Eikonal equation. Note that in our simulations, we assume that the change of refractive index along the direction of propagation (i.e., the z-axis) is negligible, and therefore, dn(z)/dz = 0. To solve the Eikonal equation, we developed an in-house numerical code implemented in the MATLAB (MATLAB R2018a, The MathWorks, Inc., Natick, Massachusetts, USA) environment. The initial conditions x_i_ and x_i_’, representing the initial position and slope of the input rays, respectively, were chosen to be *x*_*i*_ = [−1:1] mm and *x*_*i*_*’* = 0 to mimic the 2 mm collimated input laser beam that we have used in our experiments.

In practice, a finite length ultrasonic transducer will be used, and therefore, the condition dn(z)/dz = 0 will no longer be valid at the interface between the regions inside and outside the transducer since outside the transducer the refractive index is almost constant and equals the refractive index of the background. This mismatch causes refraction of the rays at the interface between the modulated medium and homogeneous medium outside the transducer. While we are interested in the behavior of light waves inside the transducer, we take into account the refraction of rays at the top and bottom interfaces by incorporating Snell’s Law into our numerical analysis.

### Experimental approach

The piezoelectric transducer (APC International, Ltd., Mackeyville, Pennsylvania, USA) is packaged with electrical leads using silver epoxy. The piezo-cylinder and wires are then coated with insulating Parylene C by using chemical vapor deposition (CVD) at room temperature (Specialty Coating Systems, Indianapolis, Indiana, USA).

The transducer is then placed on the bottom of a custom-made transparent acrylic tank. Input waveform generators (DG1022Z, Rigol Technologies, Inc., Beaverton, Oregon, USA) and a power amplifier (ENI A300, Electronics & Innovation Ltd., Rocherster, New York, USA*)* are used to feed electrical signals to the transducer.

In each experiment, we sweep the input frequency in fine steps (tenths of Hertz) to obtain resonance conditions for the ultrasonic transducer. The frequency is then fixed, and the intensities are adjusted to obtain the refractive index contrast of interest.

### Transmission imaging setup

To demonstrate the ultrasonic virtual relay imaging in transmission mode, we built a custom experimental setup consisting of a top microscope assembly and the piezoelectric transducer immersed in a water tank. The cylindrical transducer surrounds the target medium and launches standing pressure waves into it. The top microscope is used in transmission mode for imaging the *relayed* image through the ultrasonically sculpted virtual GRIN lens. The top microscope is composed of a zoom imaging lens (VZM 600i, Edmund Optics, Inc., USA), a CMOS camera (BFS-U3-51S5C-BD2, FLIR Integrated Imaging Solutions, Inc., Richmond, British Columbia, Canada*)*, and a fluorescent emission filter (MF525-39, Thorlabs, Inc., Newton, New Jersey, USA). A clear glass optical window (WG11050-A, Thorlabs, Inc., Newton, New Jersey, USA) is immersed in the medium and acts as the interface between the microscope and the imaged medium. We designed a fluorescent target object consisting of a negative transparency mask (CAD/Art Services, Inc., Bandon, Oregon, USA) of the word “CMU” (overall size: 374 × 110 µm, minimum feature size: 22 µm) overlaid on a layer of solid agar (A5306, Sigma–Aldrich, Inc., St. Louis, Missouri, USA*)* homogeneously mixed with Fluorescein dye. the sample is illuminated by a blue laser (*λ*_*light*_ = 473 nm) from the bottom.

### Lateral imaging setup

To characterize the trajectory of light passing through the modulated medium, we modified the setup to image laterally. The microscope column is rotated by 90° to image through the small lateral window opened in the wall of the transducer. We used a red laser (*λ*_*light*_ = 650 nm) modulated at the frequency of ultrasound.

The laser beam is modulated using a square wave at the same frequency of the ultrasonic waves and phase locked with the electrical signal that drives the transducer so that the laser is turned on only in the positive half period of the sinusoid wave.

### Preparation of the acousto-optic tissue phantoms

To enable lateral imaging of the formed optical beam in water, we diluted an emulsion of Intralipid 20% with deionized (DI) water to obtain a concentration of 4 × 10^−4^%. A volume of 20 μl of Intralipid is mixed in 1 L of DI water at room temperature in a Pyrex beaker. The solution is constantly stirred at 100 RPM for 1 min, until the Intralipid is uniformly mixed, and finally poured into the acrylic tank.

To create the turbid scattering medium used in this paper (0.025%), a volume of 1.25 ml of Intralipid 20% is mixed in 1 L of DI water at room temperature; the solution is successively stirred at 200 RPM for 5 min to ensure that the scattering properties of the medium are homogeneous. We note that the scattering properties, including the scattering coefficient *µ*_*s*_ and the anisotropy factor *g*, are functions of the composition of the tissue phantom as well as the operation wavelength^37,39^.

### Tissue phantoms preparation

We prepared agar-based tissue phantoms mimicking the mechanical properties of soft tissue. We mixed 2 g of powdered bacteriological agar in 100 ml of DI water at room temperature in a Pyrex beaker. We successively covered the beaker with aluminum foil to prevent evaporation, and the solution was placed on a hotplate, where the temperature was increased to the boiling point of the solution while stirring it with a magnetic stirrer at 600 RPM. When the solution was boiling, we turned off the hotplate and monitored the solution temperature while continuing to stir.

Once the solution reached 50 °C, we poured it into a rectangular mold to obtain a transparent tissue phantom made of agar 2%. As soon as the solution reached room temperature, we refrigerated it sat for 30 min at 6 °C.

To make the scattering tissue phantom, we followed the same procedure, but once the solution reached 50 °C, we added 0.5 ml of Intralipid 20% emulsion *(*I141, Sigma–Aldrich, Inc., St. Louis, Missouri, USA*)*. The solution was then stirred at 800 RPM for 5 min before it was poured into the mold and refrigerated.
